# Focus On: Magnetic Resonnance–Based Studies of Fetal Alcohol Spectrum Disorders in Animal Modals

**Published:** 2011

**Authors:** Shonagh K. O’Leary-Moore, Scott E. Parnell, Elizabeth A. Godin, Kathleen K. Sulik

**Keywords:** Prenatal alcohol exposure, fetal alcohol spectrum disorders, fetus, brain, birth defects, magnetic resonance imaging, magnetic resonance spectroscopy, diffusion tensor imaging, animal models, animal studies

## Abstract

The imaging techniques magnetic resonance imaging (MRI), diffusion tensor imaging (DTI), and magnetic resonance spectroscopy (MRS) provide valuable tools for studying brain structure and neurochemistry in fetal alcohol spectrum disorders (FASD). Although the application of magnetic resonance–based methodologies to the study of FASD in animal models is in its infancy, it already has provided new clinically relevant insights and holds significant promise to further extend our understanding of alcohol’s effects on the developing fetus.

Animal studies using magnetic resonance (MR)-based imaging technologies, including MR imaging (MRI), diffusion tensor imaging (DTI), and MR spectroscopy (MRS) provide important insight into alcohol’s early effects on the fetal brain. This article reviews how these valuable tools have been applied to the study of fetal alcohol spectrum disorders (FASD) in animal models. Compared with clinical studies of the effects of alcohol on development, using animal models allows greater control over dose, duration, and pattern of alcohol exposure. For example, research examining the effects of alcohol at different developmental stages and at various doses has helped highlight the vulnerability of the prenatal brain to very early alcohol-mediated damage. This research has important implications for clinical practice and ongoing research.

## MRI

**Figure 1:**
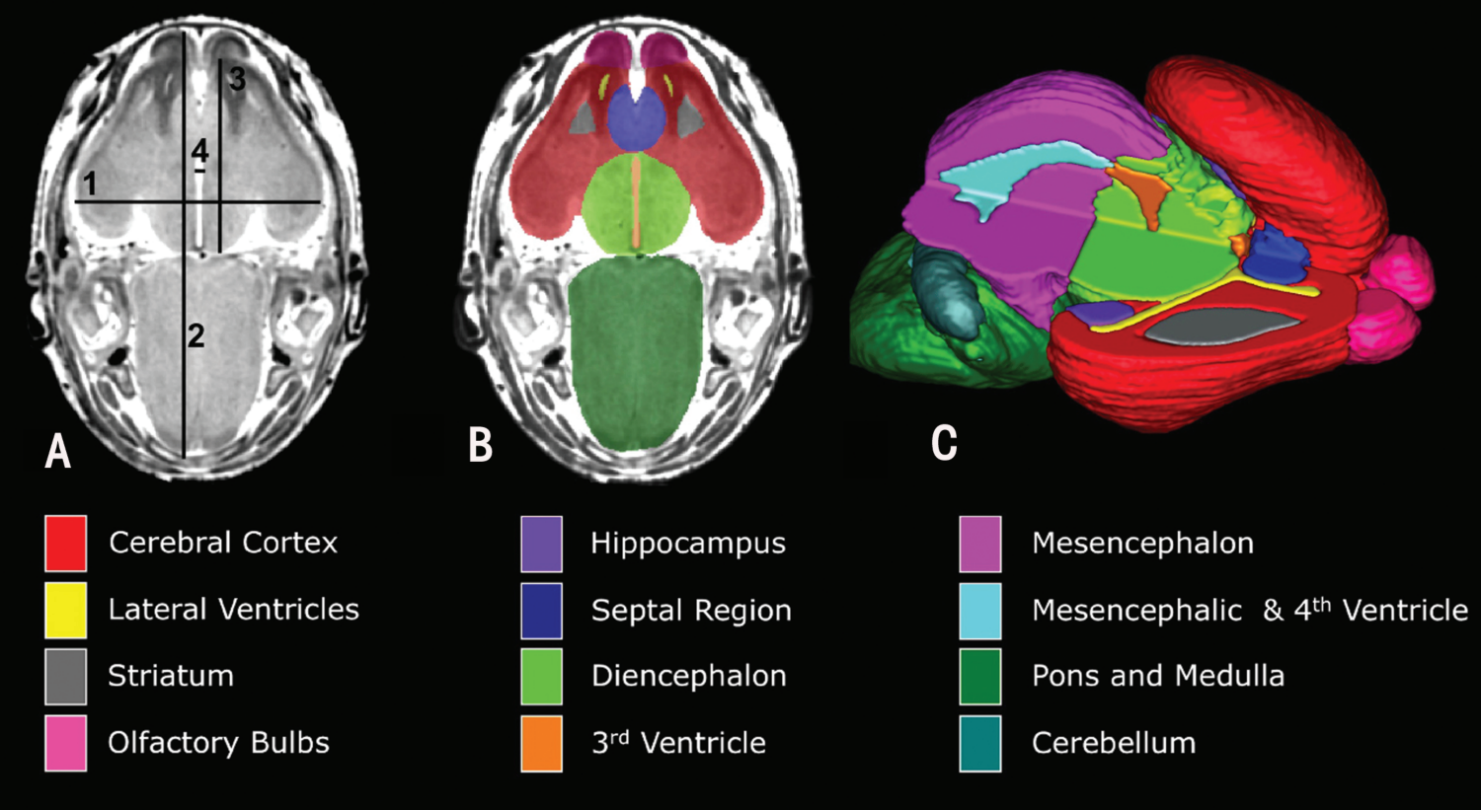
Magnetic resonance imaging (MRI) scans of mouse fetuses on gestation day 17 allow for linear measurements, regional segmentation, and three-dimensional reconstruction. **(A)** A horizontal scan is shown with lines depicting sites of linear measurements for the following: line 1, brain width (biparietal distance); line 2, midsagittal brain length; line 3, frontothalamic distance; line 4, third-ventricle width. Manual segmentation, as depicted by the color-coded regions in **(B)**, allows for subsequent three-dimensional reconstruction of selected brain regions **C. (C)** The upper right quadrant of the brain has been removed to allow visualization of the interior structures. Color codes for the segmented brain regions shown are at the bottom of the figure. The data provided by MRI is useful for the identification of gross structural brain dysmorphology and also allows the detection and characterization of subtle brain changes as follows prenatal alcohol exposure. SOURCE: Modified from Parnell et al. 2009 and Godin et al. 2010.

MRI is a noninvasive imaging technique that relies on powerful magnetic fields, radiofrequency pulses, and computer analysis to produce detailed pictures of organs, soft tissues, bone, and virtually all other internal body structures. This is in contrast to other imaging techniques that use ionizing radiation (e.g., X-rays). The application of MRI to the study of small animals has been facilitated by the development and availability of high–field strength MR systems (7 to 14 Tesla); custom radiofrequency coils (i.e., coils made specifically for imaging small animals); and the use of active-staining contrast agents, which are used to enhance the contrast of structures being viewed (reviewed by Petiet et al. 2007; Turnbull and Mori 2007). Advances in technology have allowed researchers to generate MR images with high spatial resolution, which is the ability to distinguish between two points, and sufficient contrast to allow delineation of the brain structures beneath the cerebral cortex, even in fetal mice (Petiet et al. 2008). The generation of MR scans that are isotropic (i.e., the volume elements [voxels] are uniform in all directions) allows these high-resolution images to be readily realigned and visualized in all planes simultaneously, thus facilitating accurate segmentation, three-dimensional reconstruction, and volumetric assessments of selected brain regions (see figure 1). It currently takes 2 to 3 hours to acquire high-resolution isotropic images of a mouse fetus, though technological advances promise to greatly reduce the amount of time (and expense) required.

## Studies of Alcohol-Induced Damage

In addition to providing a broad perspective on normal development, MRI also has tremendous potential to aid in explaining abnormal tissue formation. To date, in the few published reports describing MRI-based analyses of alcohol-induced defects in animal models, the brain has been the region most studied (Astley et al. 1995; Godin et al. 2010; Miller et al. 1999; Parnell et al. 2009). Future application of this technology to the study of alcohol’s effects on abnormal development in other organ systems (e.g., the heart and kidney) is also promising (e.g., Petiet et al. 2008).

Applying high-resolution MRI (also known as MR microscopy) to an established mouse FASD model, the authors have initiated a series of investigations aimed at identifying dose- and developmental stage–dependent patterns of alcohol-induced abnormalities. With the goal of identifying both mild and severe structural brain defects, and recognizing that defects at the severe end of the spectrum may not be compatible with postnatal viability in mice, the researchers selected a late prenatal stage (i.e., gestational day [GD] 17) for initial end point analyses. To date, results have been published from MRI-based analysis of defects following acute high-dose alcohol exposure (i.e., peak blood alcohol concentrations ranging from 350 to 440 mg/dl or 0.35 to 0.40 percent) on GD 7 and GD 8 (corresponding, respectively, to late in week 3 and early in week 4 of human prenatal development) (Godin et al. 2010; Parnell et al. 2009).

**Figure 2:**
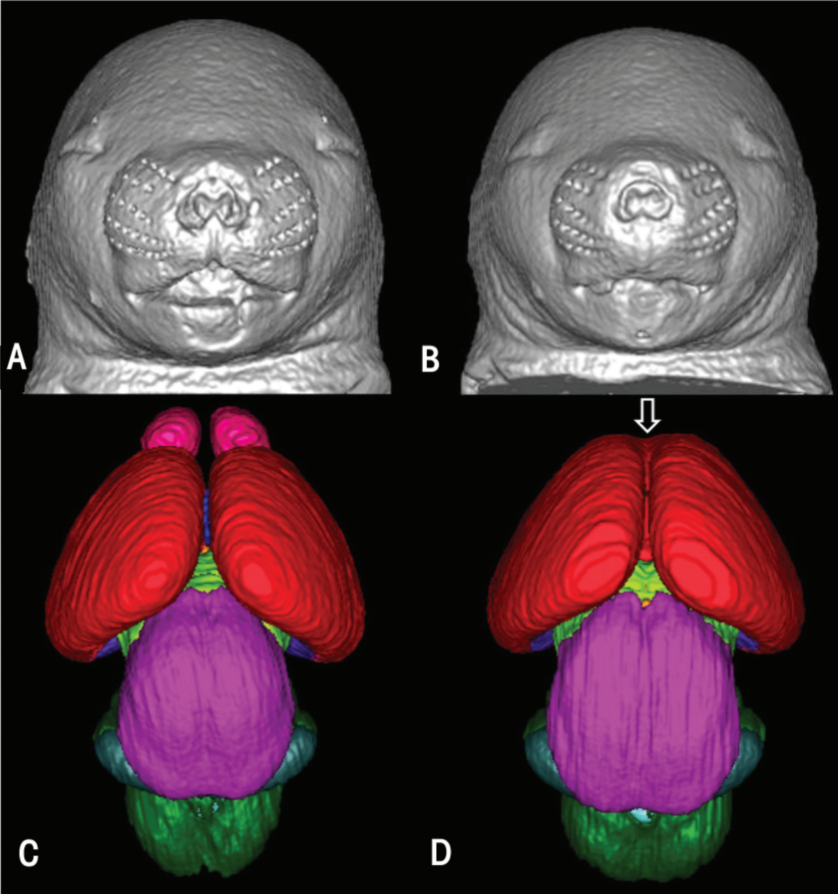
Three-dimensional reconstructions of the faces and brains of gestation day 17 control **(A** and **C)** and experimental **(B** and **D)** mouse fetuses illustrate dysmorphology resulting from acute gestational day 7 alcohol exposure. Compared with the control **(A),** the alcohol-exposed fetus **(B)** has a smaller head size, closely spaced nostrils, and an elongated/abnormal central portion of the upper lip. These facial features are characteristic of fetal alcohol syndrome. **(D)** The brain of the alcohol-exposed animal also is dysmorphic; the olfactory bulbs are absent and the cerebral hemispheres are united rostrally (open arrow). NOTES: color codes: Dark green=pons and medulla; light green=diencephalon; magenta=mesencephalon; pink=olfactory bulbs; red=cerebral cortex; teal=cerebellum. SOURCE: Modified from Godin et al. 2010.

**Figure 3:**
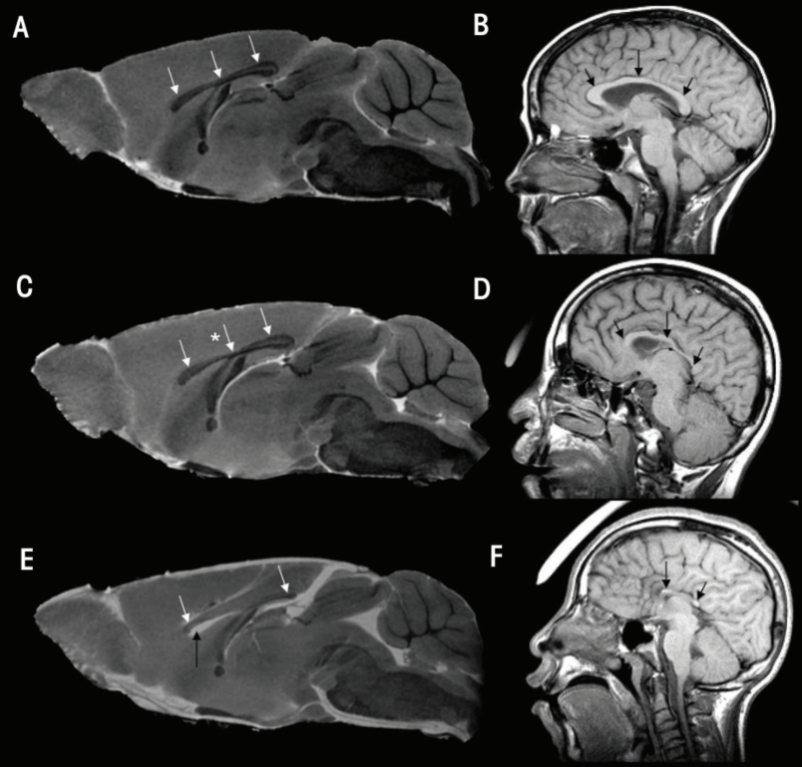
Midsagittal magnetic resonance imaging (MRI) scans of prenatal alcohol-exposed adolescent-aged mice and individuals with fetal alcohol syndrome (FAS) illustrate comparable corpus callosum dysmorphology. The normal form of the corpus callosum (CC) can be readily appreciated in a control mouse (dark structure indicated by white arrows in **(A)** and in a normal child (gray structure indicated by black arrows in **(B)**. Subsequent to prenatal alcohol exposure on gestational day 7, moderate thinning, which is particularly notable in the middle (i.e., body) of the CC (see white arrow with star) is apparent in the mouse brain shown in the MRI in **(C)**. In an MRI from a more severely affected animal shown in **(E)**, the body of the CC appears to be completely absent. The anterior and posterior aspects of the CC, although reduced in size, remain evident. These defects appear remarkably similar to those in two FAS patients (see **D** and **F**). Also notable in the most severely affected mouse is enlargement of the ventricular space (black arrow in **E**). SOURCE: The human MRIs are courtesy of S. Mattson and are modified from Mattson et al. 1994.

GD 7 alcohol exposure was shown to cause a variety of brain and facial defects that are notable on GD 17. Consistent with previous reports (reviewed by Sulik 2005; Sulik and Johnston 1982; Sulik et al. 1984), a subset of the brain defects that result from this early alcohol exposure fall within the spectrum of holoprosencephaly (HPE), a disorder characterized by abnormal median union of the right and left cerebral hemispheres and reduction in median forebrain tissues including the corpus callosum, which connects the left and right hemispheres. At the severe end of the spectrum, the tissue deficiency involves the olfactory bulbs, which may be small or absent. As shown in figure 2, three-dimensional MRI reconstructions clearly show these abnormalities in fetal mice. Additionally, as shown in this figure, MRI scans can be reconstructed to provide three-dimensional views of the facial surfaces. The facial defects caused by GD 7 alcohol exposure include abnormally close positioning of the nostrils and a long (from nose to mouth) upper lip. These facial features are consistent with those that occur in individuals with fetal alcohol syndrome (FAS). In FAS, forebrain deficiencies are a well-recognized feature, and anomalies in the corpus callosum are particularly pronounced (reviewed by Norman et al. 2009). In fact, a recent MRI study illustrated that in mice, GD 7 alcohol exposure–induced median forebrain defects also are present postnatally. As shown in figure 3, MRIs of adolescent mice prenatally exposed to alcohol show deficiencies in the corpus callosum, similar to those seen in MRIs of individuals with FAS (Mattson et al. 1994).

**Figure 4:**
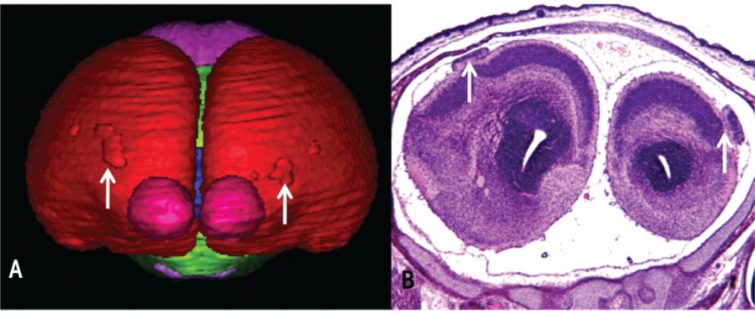
The presence of cortical heterotopias in the mouse brain following acute gestational day 7 alcohol exposure was discovered using three-dimensional reconstructions of magnetic resonance images **(A)** and confirmed using routine histology **(B)**. Notable in the frontal view shown in **A** is that both olfactory bulbs are present and separation between the cerebral hemispheres is distinct. Shown in **B**, the heterotopic cerebral cortex is intimately associated with the thin covering layer, the leptomeninges. That alcohol exposure at a time so early in gestation can result in this type of brain malformations is remarkable and clinically significant; cortical heterotopias are associated with seizure activity and epilepsy. SOURCE: Modified from Godin et al. 2010.

In addition to illustrating median facial and brain deficiencies, high-resolution MRI in fetal mice facilitated the discovery of cells in the cerebral cortex that are misplaced from their intended location as a result of GD 7 alcohol exposure (Godin et al. 2010) (see figure 4). These aberrant tissue masses (i.e., heterotopias) were first noted upon examination of three-dimensional brain reconstructions. Because MRI methodologies allow the brain tissue to remain intact, routine microscopic analysis of tissue slices (i.e., histological analysis) can be performed after imaging (see figure 4B). This histological analysis of the affected brain areas illustrated the typical features of heterotopias associated with the thin tissues covering the brain (i.e., leptomeningeal heterotopias) and allowed their classification as such. Prenatal alcohol exposure has previously been associated with heterotopias in both animal models and people with FAS (e.g., Coulter et al. 1993; Komatsu et al. 2001). The finding that alcohol-mediated damage occurring during a narrow window of very early development can result in these cerebral cortical defects is both remarkable and of significant clinical importance. Heterotopias are highly correlated with seizure disorders, and a number of human and animal studies point to prenatal alcohol exposure as a risk factor for seizure susceptibility (e.g., Bonthius et al. 2001; Sun et al. 2009).

## Quantitative Analyses

Aside from being useful for assessing structural changes following prenatal alcohol exposure, MRI is an excellent tool for conducting whole-animal–, whole-brain–, and brain-region–specific quantitative analyses. Using this application, researchers have taken regional brain-volume measurements from GD 17 fetal brains following acute alcohol exposure on each of GDs 7 and 8 (Godin et al. 2010; Parnell et al. 2009). This has provided new information regarding developmental stage–specific patterns of abnormalities. Compared with GD 7 alcohol exposure, exposure on GD 8 in mice does not result in grossly observable brain dysmorphology but causes volume reductions in selected brain regions including the olfactory bulbs, hippocampus, and cerebellum, as well as enlargement of the spaces within the brain (i.e., ventricles) (Parnell et al. 2009). In addition to allowing volumetric assessments, the future development and application of sophisticated image analysis techniques adapted for rodents promises to facilitate identification of alcohol-induced regional brain shape changes and, thus, to provide additional clues regarding alcohol’s deleterious effects on the developing brain.

Although not as “comprehensive” as volumetric analyses, linear measures are readily made from individual MRI scans (see figure 1) and also are useful for detecting patterns of effects following discrete periods of alcohol exposure. This is exemplified by the finding of reduced frontothalamic and brain-width measures in fetal mice following GD 7 alcohol exposure (Godin et al. 2010), whereas GD 8 alcohol exposure was found to reduce transverse cerebellar diameter and to increase third-ventricle widths (Parnell et al. 2009). These data confirm and extend findings from human prenatal ultrasound studies (Kfir et al. 2009) and enhance the potential for prenatal FASD diagnosis.

## DTI

DTI, an MRI technique that holds additional promise for elucidating alcohol’s adverse effects on central nervous system (CNS) development, allows visualization of the pattern and integrity of CNS fiber tracts and is based on identifying the rate and direction of water diffusion through the tissue. This technique capitalizes on the fact that diffusion is more isotropic in cerebral spinal fluid and cell bodies but greater in one direction than the others (i.e., anisotropic) along the projections of nerve cells (i.e., axons) that comprise fiber tracts. Even in the absence of gross structural brain malformations, DTI can detect microstructural changes within the fiber tracts.

**Figure 5:**
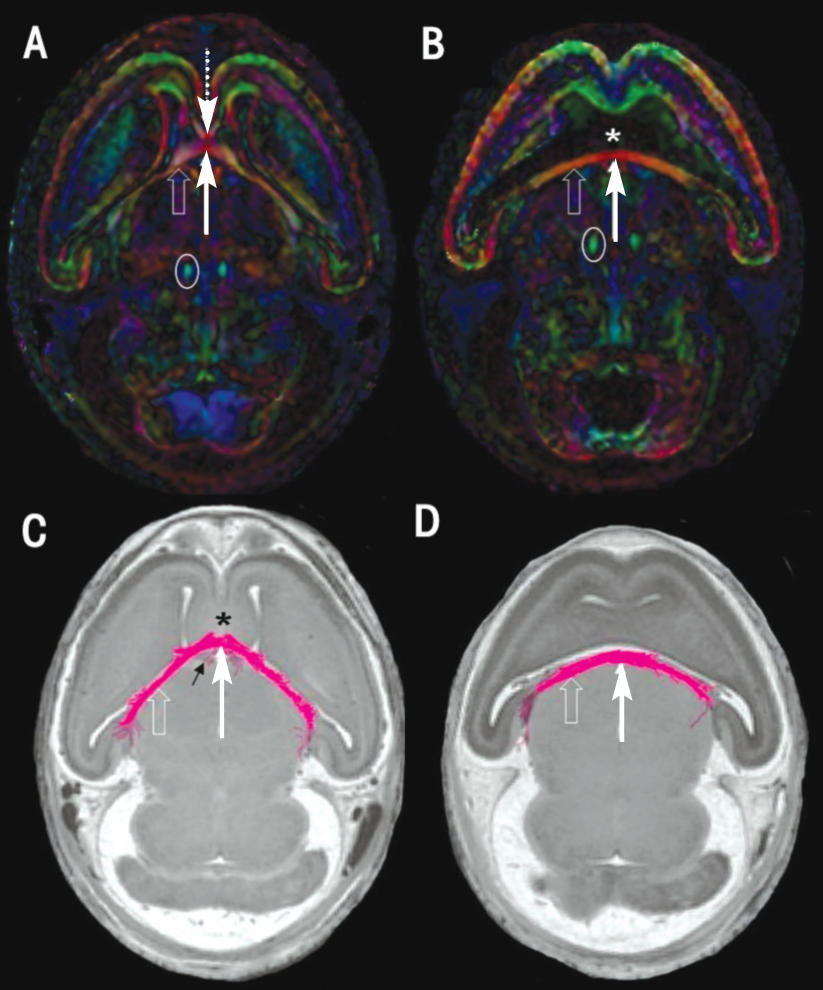
Directionally encoded color maps **(A** and **B)** and fimbria/fornix fiber tract reconstructions **(C** and **D)** from a control **(A** and **C)** and a gestational day 7 alcohol-exposed mouse fetus **(B** and **D)** are shown. The directionality of the brain fiber tracts in the color-coded maps are indicated as follows: red, left/right; blue, inferior/superior; and green, anterior/posterior. In the control **(A)**, the corpus callosum (dashed arrow), fimbria (open arrows), fornix (solid arrow), and fasciculus retroflexus (circled) are evident. In the affected fetal brain, the lateral ventricles are united in the midline (star) and the corpus callosum is not present, as evidenced by the absence of red-coded fibers. Anomalies of the fimbria and fornix also can be appreciated in the color-coded maps and three-dimensional fiber-tract reconstructions **(C** and **D)**, (magenta fibers). In the control fetus **(C)**, the fibers of the fimbria/fornix project from the hippocampus forward toward the septal region (star) and then inferiorly forming the columns of the fornix (black arrow). In contrast, in the alcohol-exposed fetus **(D)**, the fimbria and fornix appear to be significantly shortened, flattened, and thickened, whereas the septal region and fornix columns are completely absent.

Researchers have reported a number of DTI studies of rodent brains (reviewed by Mori and Zhang 2006). Imaging mouse fetuses as young as GD 12, Zhang and colleagues (2003) showed that even before axons develop their insulating myelin sheaths, fiber orientation within the developing brain can be identified using DTI. The fiber orientation data can be depicted in color-coded maps, examples of which are shown in images of a control and alcohol-exposed GD 17 mouse brain in figures 5A and 5B, respectively. In these images, the red, blue, and green colors indicate fibers running in left/right, inferior/superior, and anterior/posterior directions, respectively. Among the fiber tracts that are evident in the normal fetal brain are the corpus callosum; the fimbria, a band of white matter along the edge of the hippocampus; the fornix, a bundle of fibers that connects the hippocampus on either side of the brain and then projects down and back toward the thalamus; and the fasciculus retroflexus, which consists of major output fibers of the forebrain. DTI data also can be depicted by using specialized processing software to render three-dimensional reconstructions of the fiber tracts, examples of which are shown for the fimbria/fornix of a GD 17 control and alcohol-exposed fetus in figures 5C and 5D, respectively.

In the FASD research arena, DTI has been applied to define white-matter anomalies in the human whole brain (e.g., Fryer 2009; Lebel et al. 2008, 2010; Sowell et al. 2008). The majority of these studies have focused primarily on examination of the corpus callosum. In both children and young adults who were prenatally exposed to alcohol, researchers have reported alterations in measures of diffusivity involving this major fiber tract (e.g., Fryer et al. 2009; Lebel et al. 2008, 2010; Li et al. 2009; Ma et al. 2005; Sowell et al. 2008; Wozniak et al. 2006, 2009). O’Leary-Moore and colleagues (2010) currently are using DTI in animals to extend the MRI-based FASD studies described above. Following GD 7 alcohol exposure, the researchers have found deficits in a number of major fiber tracts including the corpus callosum and fimbria/fornix in their mouse model (see figure 5B and D). It is anticipated that these ongoing ex vivo DTI studies will yield a wealth of information and that technological advances will reduce the time required for small-animal imaging and provide the potential to extend the analyses to live specimens.

## MRS

MRS is a technique that allows the noninvasive assessment of a number of neurochemicals in the brain and detection of subtle changes in brain biochemistry in the absence of gross dysmorphology. Based on the same principles as MRI, MRS suppresses water signals (which typically are exploited in anatomical imaging) and provides the ability to assess neurochemicals in localized regions of the brain (Moore and Galloway 2002). It appears that MRS-based neurochemical data may be useful as a biomarker for prenatal alcohol exposure.

Among the neurochemicals that can be measured using MRS, the three most readily assessed are *N*-acetyl-aspartate (NAA), a marker of neuronal integrity that is reduced in a myriad of disease states; choline, an essential nutrient necessary for the synthesis of acetylcholine and the cell membrane constituent phosphatidylcholine; and creatine, a high-energy phosphate important in maintaining cellular energy–dependent systems. Studies in humans and non-human primates have shown changes in each of these neurochemicals following prenatal alcohol exposure (Astley et al. 1995, 2009; Cortese et al. 2006; Fagerlund et al. 2006). However, the direction of change and the brain region dependency of these effects have varied, possibly as a result of different stages of development of the subjects at the time of analysis and variations in alcohol dose and exposure times between study groups (Astley et al. 2009; Cortese et al. 2006; Fagerlund et al. 2006). Other neurochemicals also are clinically identifiable, but they are less frequently reported and often require the use of higher-field-strength magnets (4 Tesla) or specialized techniques for their measurement and quantification (Moore and Galloway 2002).

**Figure 6:**
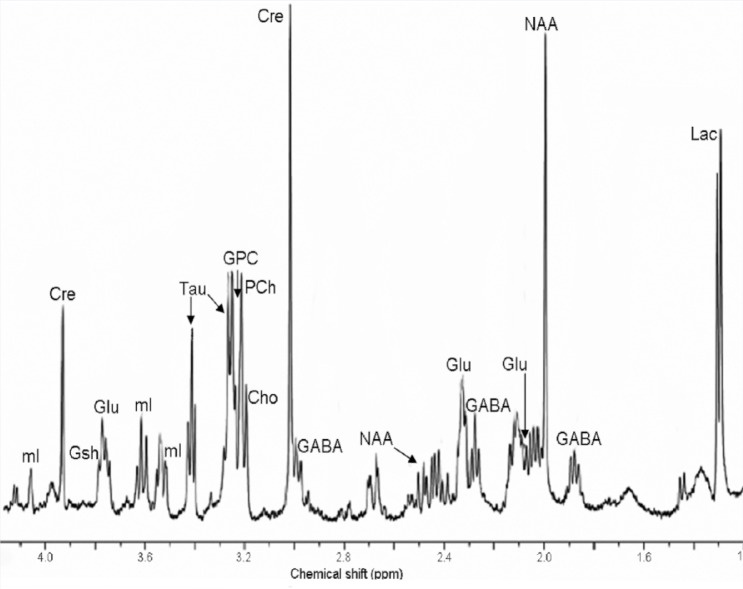
A spectra acquired from an adult rat striatum using high-resolution magic-angle spinning (HR-MAS) magnetic resonance spectroscopy (MRS) illustrates the spectral resolution obtained using this technique. Neurochemicals that often are measured clinically are readily identified, including *N*-acetylaspartate (NAA), which is reflective of neuronal density and is involved in myelin synthesis and osmoregulation; creatine (Cre), a high-energy phosphate; and choline (Cho), which is indicative of cell-membrane integrity and acetylcholine synthesis. The high-resolution scan also allows identification of phosphorylcholine (Pch), a membrane phospholipid precursor and glycerophos-phorylcholine (GPC), a membrane phospholipid breakdown product; and compounds that overlap with and make up the choline peak in lower-resolution scans. Neurochemicals that play important roles in excitatory and inhibitory neurotransmission such as glutamate (Glu) and γ-aminobutyric acid (GABA) also are identifiable as are antioxidants such as taurine (Tau), glutathione (Gsh), and the glial marker/osmolyte myo-Inositol (ml). SOURCE: Modified from O’Leary-Moore et al. 2008.

Facilitated by the availability of high-resolution MRS techniques such as high-resolution magic-angle spinning (HR-MAS) MRS, researchers have initiated MRS-based analyses employing rodent FASD models to elucidate developmental stage– and region-dependent neurochemical changes. HR-MAS MRS provides neurochemical information from intact ex vivo tissue punches from multiple localized brain regions and allows the assessment of more neurochemicals than is possible using conventional MRS in clinical populations. Figure 6 shows a typical HR-MAS MRS neurochemical spectrum. Here, each neurochemical is identified by its unique chemical shift position on the horizontal axis. The concentration of each neurochemical is proportional to the area under its respective peak(s). Applying this technique, O’Leary-Moore and colleagues (2008) showed that neonatal alcohol exposure in rats (at a time corresponding to the human third trimester) significantly reduced levels of NAA and taurine (an inhibitory neuromodulator and antioxidant) in the cerebellum and striatum; elevated levels of myoinositol, a marker for neuronal support cells (i.e., glial cells) in the cerebellum; and, in females, reduced cerebellar glutamate levels. The lowered NAA and glutamate levels may indicate that significant cell loss occurred as a result of the alcohol exposure. This is consistent with previously reported cerebellar cell number reductions resulting from alcohol exposure during the neonatal period (reviewed by Green 2004). Whether these reductions in number are directly related to diminished antioxidant capacity, as indicated by low taurine levels, remains to be determined. In this regard, it is notable that a number of studies have shown the potential of antioxidants to reduce alcohol’s disruptive effects on development (reviewed by Cohen-Kerem and Koren 2003).

## Conclusions

The continued application of MR-based technologies to the study of FASD in animal models promises to greatly facilitate the definition of the full spectrum of alcohol-induced birth defects along with their developmental stage and dosage dependency. Recognition of the entire range of consequences is essential for accurate diagnosis and prevention. With respect to the latter, MRI-based rodent studies have highlighted the vulnerability of the brain to alcohol-mediated damage resulting from alcohol exposure at developmental stages that occur in humans prior to the time that pregnancy is typically recognized, underscoring the importance of pre-pregnancy counseling. In addition to the ex vivo analyses that have been conducted to date, evolving MRI technologies are expected to allow longitudinal studies of both the brain and behavior in individual animals. Overall, these MR-based animal studies are expected to provide data that will inform clinical practice and ongoing research.
